# Gait quality in prosthesis users is reflected by force-based metrics when learning to walk on a new research-grade powered prosthesis

**DOI:** 10.3389/fresc.2024.1339856

**Published:** 2024-02-02

**Authors:** Kinsey R. Herrin, Samuel T. Kwak, Chase G. Rock, Young-Hui Chang

**Affiliations:** ^1^ Woodruff School of Mechanical Engineering, Georgia Institute of Technology, Atlanta, GA, United States; ^2^ Institute for Robotics and Intelligent Machines, Georgia Institute of Technology, Atlanta, GA, United States; ^3^ School of Biological Sciences, Georgia Institute of Technology, Atlanta, GA, United States

**Keywords:** gait, wearable, robotics, sensors, prosthetics, outcome measures

## Abstract

**Introduction:**

Powered prosthetic feet require customized tuning to ensure comfort and long-term success for the user, but tuning in both clinical and research settings is subjective, time intensive, and the standard for tuning can vary depending on the patient's and the prosthetist's experience levels.

**Methods:**

Therefore, we studied eight different metrics of gait quality associated with use of a research-grade powered prosthetic foot in seven individuals with transtibial amputation during treadmill walking. We compared clinically tuned and untuned conditions with the goal of identifying performance-based metrics capable of distinguishing between good (as determined by a clinician) from poor gait quality.

**Results:**

Differences between the tuned and untuned conditions were reflected in ankle power, both the vertical and anterior-posterior impulse symmetry indices, limb-force alignment, and positive ankle work, with improvements seen in all metrics during use of the tuned prosthesis.

**Discussion:**

Notably, all of these metrics relate to the timing of force generation during walking which is information not directly accessible to a prosthetist during a typical tuning process. This work indicates that relevant, real-time biomechanical data provided to the prosthetist through the future provision of wearable sensors may enhance and improve future clinical tuning procedures associated with powered prostheses as well as their long-term outcomes.

## Introduction

There is mixed evidence on the benefits associated with the use of powered prosthetic feet compared with passive feet (1, 2). Some studies reported increases in preferred walking speed with the use of powered feet (3), while others found no differences in speed in the lab or in daily life (4). Some studies have shown benefits over passive prosthetic feet for select user groups in regard to metabolic cost (3, 5), while others found no difference (4). Some studies have shown improvements in symmetry (6), while others showed increased asymmetry with the use of a powered foot (7). Some studies reported improvements in pain scores with the use of a powered prosthesis (8), while others noted subject-specific reports of an increase in pain (4). While the evidence is indeed conflicting, the outcomes reported in these studies have important implications for a patient's overall quality of life. The self-selected walking speed is known to have a heavy influence on a patient's quality of life and independence (9), metabolic cost influences a patient's mobility ability level (10, 11), and gait asymmetries are tied to longer-term secondary issues such as osteoarthritis and low back pain (12), which also influence quality of life. Efforts have been made to understand the unequal effectiveness of these prosthetic feet among patients, with some evidence pointing toward the lack of coordination between the human body and the device (1, 13), as well as the limitations in transferring energy from the prosthetic foot to the center of mass and the lack of proper tuning (13). Despite the mixed evidence on powered prosthetic feet, for any benefits to be realized during the use of a powered prosthetic foot, it must be appropriately custom-tuned to the individual with the amputation such that they are comfortable, the gait is as normative as possible, and it will allow for a long-term successful wear.

In clinical practice, prosthetists have a significant influence on the outcomes for individuals with lower limb amputation (14). Powered devices by nature are complex (5), and in research settings, it can take an engineer several hours to manually tune a device for each individual (14, 15). Tuning is an applied skill in which the prosthetist uses an observational gait analysis along with patient-reported feedback to customize the parameters selected for a specific individual (5); tuning also functions as an iterative process requiring collaboration between the patient and the prosthetist. Tuning in both clinical and research settings is subjective, time-intensive, and the standard by which the prosthetist tunes can vary depending on the experience levels of both the patient and the prosthetist (16). Furthermore, if the treating prosthetist is inexperienced with the technology, the tuning of powered, commercially available feet may require the involvement of a manufacturer representative with more extensive device knowledge (5), which may introduce barriers to initial access for the advanced technology and/or barriers to long-term functional gains in the event of changes in patient functional status. In the face of these challenges, a better understanding of the biomechanics underlying the tuning process could help clinicians identify specific areas to focus on, while also providing researchers with relevant data to study.

It is with this motivation that we studied the tuning process and subsequent metrics of gait quality associated with a research-grade powered prosthetic foot in both clinically tuned and untuned conditions. In this study, we investigate the ability of eight metrics of gait quality, as described below, to distinguish between a tuned and untuned powered prosthetic foot, with the goal of identifying the metrics capable of distinguishing between what is clinically known to be good and poor gait quality. We hypothesize that the metrics with a more comprehensive assessment of gait will have the highest probability of detecting the differences following tuning of a research-grade powered prosthetic foot, given the known influence of prosthetic componentry on the functional walking performance of a patient (17, 18).

## Methods

### Participants

The inclusion criteria for participants with amputation were as follows: aged between 18 and 69 years, at least 12 months post-transtibial amputation, classified as a K3–K4 walker, capable of walking with a prosthesis without assistive devices, and not using a solid ankle, cushion heel (SACH) foot as clinically prescribed. The participants were excluded if they met any of the following criteria: presence of dementia or inability to give informed consent, significant loss of hip, knee, or ankle joint motion, history of dizziness and/or balance problems, and currently pregnant.

### Experimental procedures

**Figure 1:**
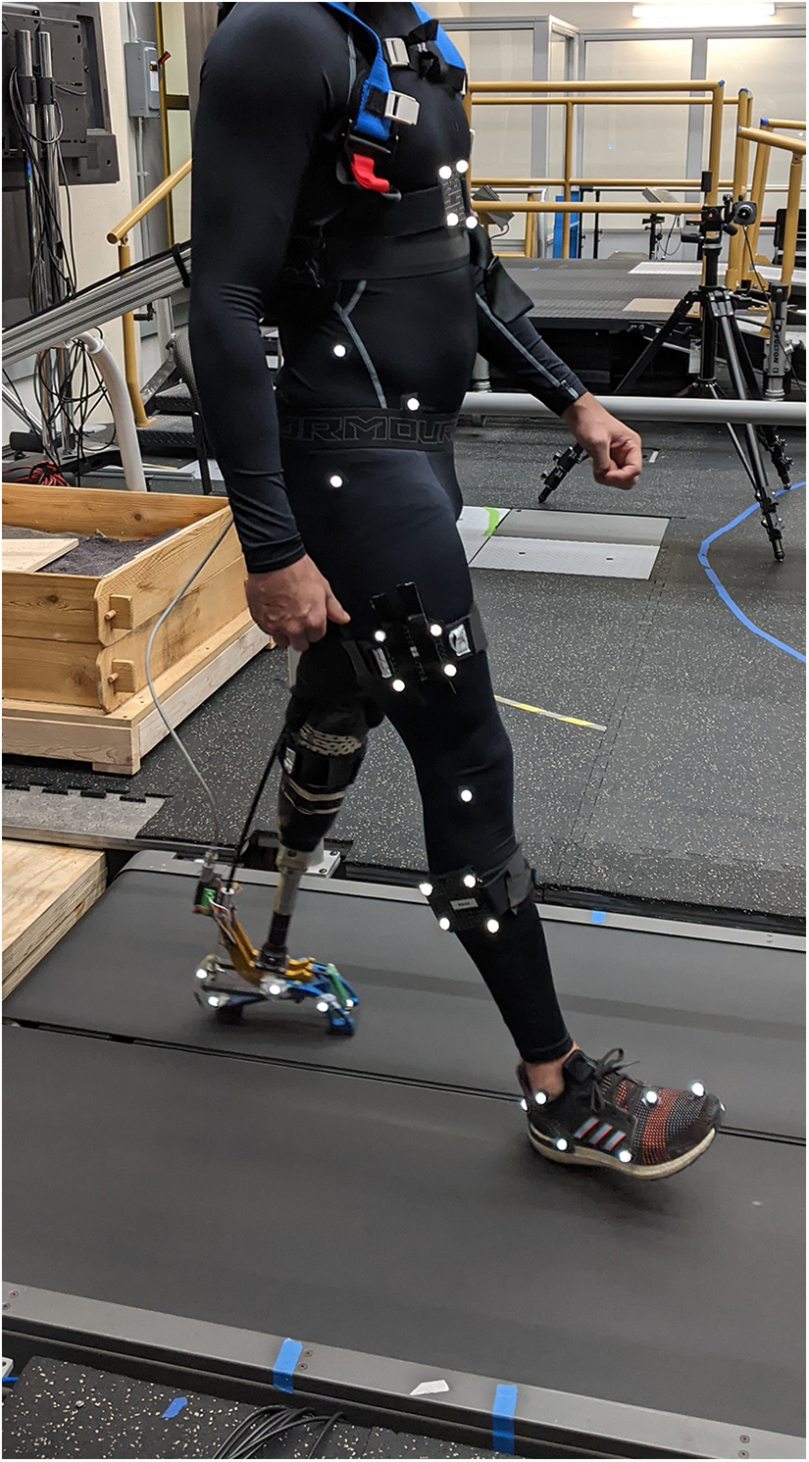
Showing experimental setup inclusive research-grade powered prosthetic foot, motion capture marker placement, and instrumented Bertec treadmill.

The participants were fit with a commercially available tethered research-grade powered prosthetic foot (Humotech PRO-001, Humotech, Pittsburgh, USA) as described in (19–21) by a certified prosthetist. The foot was attached to the participant's current socket and aligned until both the user and prosthetist were satisfied with the alignment and motion in all planes, similar to the methods described by Ingraham et al. (22). While standing, the participants were instructed to push into the device to gain comfort and familiarity with the stiffness level of the foot prior to any tuning or walking. Retroreflective markers were placed on anatomical landmarks using a modified Helen Hayes marker set (23) as shown in Figure 1. The subjects then walked on a split-belt treadmill at a predetermined speed (1.0 m/s) while lower limb and trunk biomechanics were collected, first wearing their clinically prescribed passive prosthetic foot for approximately 1 min and switching to the powered prosthetic foot. The speed of 1.0 m/s was selected as it falls within the previously reported values for transtibial prosthesis users during treadmill walking (24). The walking trial in the passive prosthetic foot provided the opportunity for the participants to gain comfort walking on a treadmill at the selected speed, and the data are solely intended for the purpose of comparison. Lower body and trunk kinematics were collected using a 36-camera motion capture system (Vicon, Centennial, CO; Visual 3D, C-Motion, Germantown, MD). Ground reaction forces were recorded from under each foot using an instrumented split-belt treadmill (Bertec, Columbus, OH, USA) (25, 26). Synchronized, optical video data were also recorded in both the sagittal and frontal planes (Vicon Bonita cameras). During the walking trials with the powered prosthetic foot, the study team, which included an experienced certified prosthetist, iteratively tuned the foot according to current clinical practice methods that include an observational gait analysis and patient feedback, similar to the methods described by Ingraham et al. (5, 22). The participants were encouraged to try to maintain equal step lengths and stance times throughout the tuning procedure when possible. We used the default settings of the device with a correction for the body weight of the individual participant as the untuned baseline condition. This trial was captured prior to any tuning and was deemed the untuned baseline condition. Following the baseline condition, we iteratively tuned the features of dorsiflexion stiffness, dorsiflexion and plantarflexion range of motion, timing of plantarflexion torque, and magnitude of plantarflexion torque. The participants were encouraged to share any information with the team at each parameter change. If a particular feature of the foot caused discomfort, it was immediately re-tuned in the next parameter change trial. For example, if the timing of plantarflexion torque was being tuned and the participant reported feeling uncomfortable due to dorsiflexion stiffness, this was immediately tuned in the next trial. Following each parameter tuning change, the participant was given approximately 30 s to acclimate to the change, and a 15 s walking trial was recorded. Due to the iterative nature of the tuning process, it was possible for some parameter changes to unintentionally have a negative impact on the participant's gait and/or comfort. It was also possible for a single parameter value to be trialed more than once by a participant, and features such as dorsiflexion stiffness and the timing of the plantarflexion torque may have been revisited multiple times.

There were 15 tunable parameters as described in Table 1 that were manipulated with an average of 8 ± 1 of the 15 parameters altered for each participant. All participants were able to walk in the baseline condition. An average of 24 ± 5 iterative parameter changes were required before the tuning process of the powered prosthetic foot was deemed complete by the full research team, which included the participant. The baseline and tuned parameter values are detailed in Supplementary Table S1 for each participant. The tuning process proceeded until the participant's gait was noted to be visually acceptable and the participant reported feeling comfortable, similar to other studies that have relied on user preference for the fine-tuning of powered foot parameters (4, 5, 22). Participant feedback was critically valued in the tuning process during this study. Visually, the team continually assessed for prosthetic gait deviations (i.e., vaulting, early heel rise, excessive varus/valgus, swing phase clearance, swing phase whips, controlled plantarflexion at heel strike, appropriate positioning of the foot at heel strike), changes in spatiotemporal symmetry, perceived congruity of the device with the participant and limb positioning during the mid and late stance, and appropriateness of the plantarflexion torque timing with each parameter change similar to (15). Finally, the tuning process concluded when the study team determined that no other tuning changes would further improve the participant's gait visually or in comfort level and the participant confirmed that they felt most comfortable with the selected parameters; this trial was deemed the tuned condition.

**Table 1:** Descriptions of the 15 tuning parameters that were altered during the tuning process.

Parameter	Parameter description
MaxDorsi (deg)	Changes the peak angle (in degrees) at which the device will exert peak torque.
MaxPlantar Dorsi (deg)	Changes the starting angle (in degrees) of the walking cycle.
MaxPlantar Plantar (deg)	Changes the angle (in degrees) of the end point of the plantarflexion part of the walking cycle.
HorizShift (deg)	Shifts the graph and all control points along the ankle angle axis (in degrees).
Shape Dorsi	Changes the control point for the dorsiflexion curve, the control point will move perpendicular to the straight line between the dorsiflexion starting and end points. Changes whether work done is positive or negative.
Shape Plantar	Changes the control point for the plantarflexion curve, the control point will move perpendicular to the straight line between the plantarflexion starting and end points. Changes whether work done is positive or negative.
Max Torque (Nm)	Changes the peak torque (in Newton-meters) value of the control curve during a walk cycle.
Min Torque Dorsi (Nm)	Changes the starting torque (in Newton-meters) of the walking cycle.
Min Torque Plantar (Nm)	Changes the torque (in Newton-meters) of the end point of the plantarflexion part of the walking cycle.
Toe_Clear (deg)	Angle targeted at the ankle during swing state.
Tau_thresh	The minimum requirement (in Newton-meters) that signals to the walking controller the beginning of a step. Raising the value will make steps start later because of the higher load requirement, and lowering the value will make steps start sooner but also be susceptible to false positives due to signal noise.
Plantar Trans Tor	Like tau_thresh, it is the minimum threshold of ankle torque (in Newton-meters) required to pass into the plantarflexion state.
Plantar Trans Pow Now	The minimum power output (ankle torque * ankle angle velocity) in the current timestep required to pass into the plantarflexion state.
Plantar Trans Pow Previous (W)	The minimum power output (ankle torque * ankle angle velocity) in the previous timestep required to pass into the plantarflexion state.
Plantar Trans T (sec)	The minimum length of time (in seconds) required to be in dorsiflexion before the transition to plantarflexion can be allowed.

### Data processing and gait metrics

The Visual 3D software was used to filter data (fourth-order Butterworth with cut-off frequencies at 6 Hz for the force and marker data), as well as to calculate inverse kinematics and kinetics. Data were exported to MATLAB (R2022b, Mathworks, Inc.) for additional processing. To compare between the untuned and final-tuned condition, metrics that were calculated included unified deformable ankle–foot (UDAF) peak power, leg work, impulse symmetry in the vertical and anterior–posterior planes, positive ankle work, limb-force alignment (LFA), the gait quality index (GQI), the prosthetic observational gait score (POGS), and lateral sway. These metrics were selected to provide a broad range of perspectives in the field of biomechanics and gait analysis with some metrics more biomechanically comprehensive, some more computationally intensive, and some more simple in approach. Below is a description of how we defined these gait metrics.

### Unified deformable ankle–foot positive work and peak power

The unified deformable (UD) method for determining joint power, particularly ankle power, has become a preferred method for determining the mechanics of a prosthesis. In this study, ankle work and peak power for both the prosthetic and sound sides were calculated using the unified deformable segment model method and normalized to participant body mass. A key benefit of the UD method is that it does not require the determination of a specific ankle joint, which is typically required in classical inverse dynamics equations. This makes it a useful method for characterizing the mechanics of a prosthesis, which lacks a specific axis of rotation. UDAF power was calculated in Visual3D for both the prosthetic and sound limbs, and the positive power near the end of stance (i.e., push-off) was integrated to determine push-off work. More details on the UD calculation can be found in (27).

### Leg work

Leg work refers to the positive mechanical work done on the center of mass over a single stride and calculated using inverse dynamics based on the ground reaction forces collected on the instrumented treadmill with a custom code in Matlab, similar to the one used by Selgrade and colleagues (28), and was normalized to the body mass of the participant.

### Impulse symmetry

Impulse symmetry was calculated using the following equation (29):

$$\mathrm{Impulse}\;\mathrm{symmetry}=\frac{\mathrm{Impuls}e_{\mathrm{Sound}}-\mathrm{Impuls}e_{\mathrm{Prosthesis}}}{\frac{1}{2}(\mathrm{Impuls}e_{\mathrm{Sound}}+\mathrm{Impuls}e_{\mathrm{Prosthesis}})}\;100\text{\%}$$

A value of zero is indicative of complete symmetry between the prosthesis and sound side limbs. Positive values are indicative of larger impulses on the sound side, whereas negative values are indicative of larger impulses on the prosthesis side.

We show the force impulse symmetry index calculated from both the vertical and anterior–posterior components of ground reaction force.

### Limb-force alignment

The LFA is a novel metric that is determined by dividing the angle of the sagittal plane ground reaction force by the angle of the trailing limb (30) at the time of peak force production as follows:

Limb-ForceAlignment=TrailingLimbAngle/GRFAngle

A score of 100% is equivalent to complete the alignment between these two vectors. The alignment of these vectors is relevant because it allows for reduced joint moments and muscle forces and therefore a more effective mechanical advantage given the more efficient force application directed along the leg (31–34). The angle of the ground reaction force was calculated as the angle of the force vector in the sagittal plane at the time of peak anterior force. Vertical angles were set equal to zero, and thus greater angles are more anteriorly directed. Trailing limb angle was calculated at the same time point and defined as the sagittal plane angle of the fictional segment connecting the center of pressure to the retroreflective marker over the greater trochanter of the femur, as in (30), with 0 degrees indicative of vertical orientation and values greater than 0 degrees indicative of the greater trochanter being more anterior to the center of pressure.

### Gait quality index

The GQI was calculated using the method reported in (35) and provides a summary score of gait quality, which encompasses kinematics, kinetics, and spatiotemporal measures, with scores closer to zero indicative of a more normative gait quality. The GQI is an average of subscores calculated from a temporal-spatial quality index, a kinematic quality index, and a kinetic quality index, with scores closer to zero indicative of a normative gait pattern. The temporal-spatial quality index is composed of velocity, cadence, bilateral step and stride lengths, and step width, all normalized to height with the exception of cadence. The kinematic quality index is composed of the sagittal and frontal plane measures of the trunk, pelvis, and hip and the sagittal plane measures of the knee and ankle, and the kinetic quality index is composed of hip moments in the frontal and sagittal planes and knee and ankle moments in the sagittal plane.

The control population used for the comparison in the GQI calculation consisted of nine able-bodied individuals who were matched in terms of age, weight, and height (age 39.3 ± 16.8 years, weight 78 ± 12.8 kg, height 1.7 ± 0.1 m). These individuals provided written, informed consent to participate in a prior lab trial under the same protocol as the participants with amputation herein. Because the powered prosthetic foot did not have a conventional ankle joint, we used the same unified deformable segment model method (27) described above for the ankle moment calculations on both limbs for the control population. More details on the GQI calculation can be found in (35).

### Prosthetic observational gait score

The prosthetic observational gait score (POGS) was calculated for the prosthetic side using the method reported in (36) with a score of 32 indicative of a poorer quality gait and a score of 0 indicative of a better gait quality. There are 16 aspects of a patient's gait that are scored as part of the POGS calculation including arm swing, vaulting in stance, lateral and anterior/posterior trunk lean, hip extension and flexion in stance and swing, knee extension in stance, knee flexion in terminal stance, initial swing and terminal swing, step symmetry, first ankle rocker, foot rotation at initial contact, width of the base of support, circumduction, and whips. The video footage of participants walking in the robotic foot was blinded and scored by a clinician with the aid of an on-screen digital goniometer for improved accuracy. The passive prosthetic foot condition was not blinded due to the visual nature of the metric, and again, it is provided for visual reference only.

### Lateral sway

The lateral sway was calculated for each stride by taking the difference in the maximum and minimum values of the mediolateral trajectory of a sternal chest marker cluster in the coronal plane as in the following equation:

Lateralsway=Maximumposition-Minimumposition

Paired *t*-tests were completed with statistical software (Minitab 19.2020.1, State College, PA) to compare the impact of tuning on the gait metrics between the untuned and tuned conditions. We defined alpha 0.05 throughout our analysis. Metrics were additionally calculated for the clinically prescribed passive foot condition. Due to the numerous differences in the two styles of feet (i.e., wear/use time, inconsistent shoe use, foot length, tethered capacity), formal statistical comparisons are not provided and are shown in the analysis that follows for visual reference only.

## Results

### Participants

Two females and five males (age 37.0 ± 10.5 years, weight 81 ± 8.8 kg, height 1.8 ± 0.1 m) with unilateral transtibial amputation were recruited for this study and provided written, informed consent prior to participating in this study according to the Georgia Institute of Technology Institutional Review Board protocol H17290. The average time since amputation for all subjects was 4 years, 7 months ± 2 years. All participants wore total surface bearing socket designs with either pin, suction, or vacuum suspension systems. All participants had a passive, dynamic-response, and energy storage and return clinically prescribed prosthetic foot. Six of the seven participants were amputated on the left side.

### UDAF positive work, peak power, and leg work

**Figure 2:**
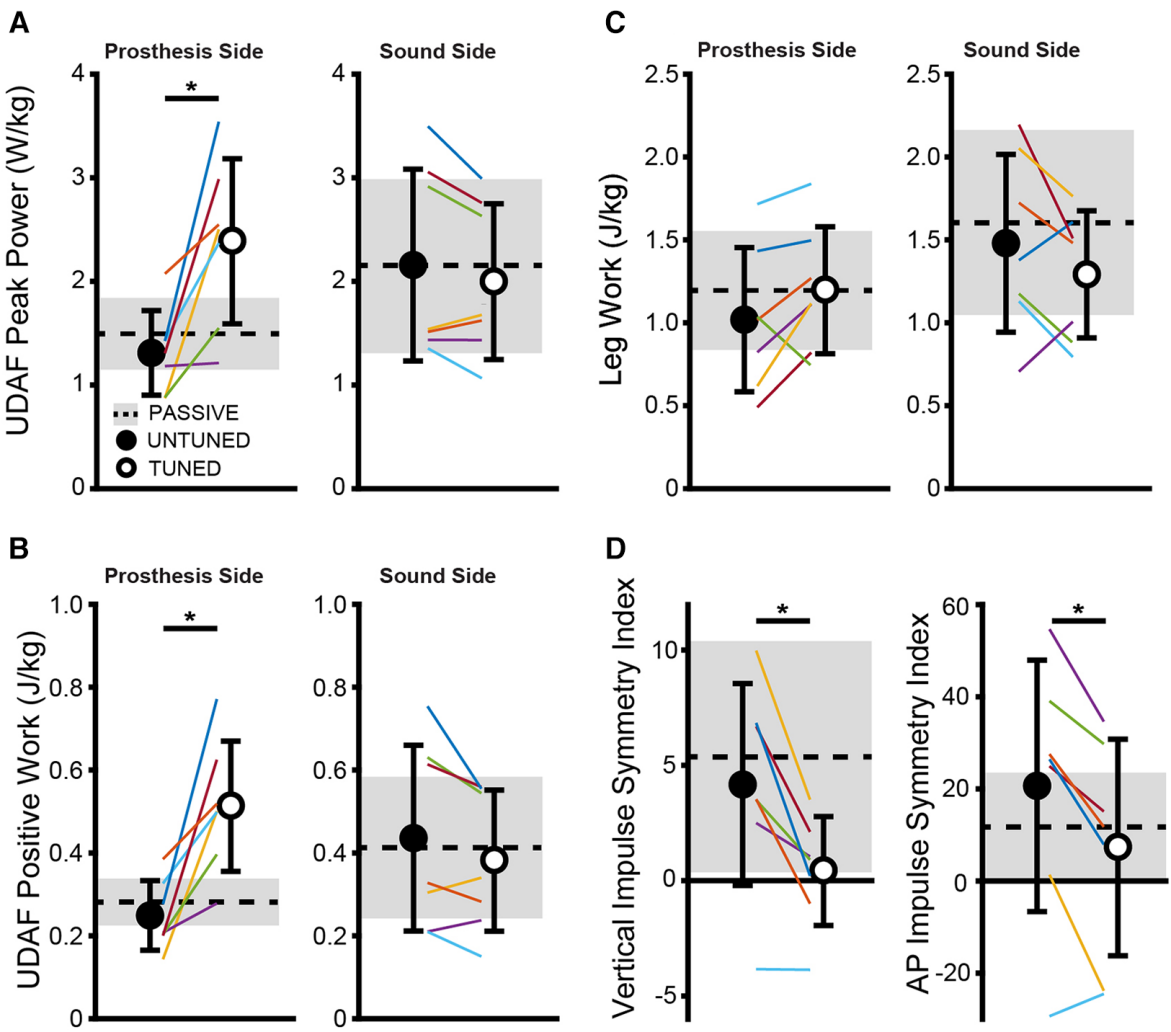
The tuned condition shows significantly greater ankle peak power (**A**) and positive work (**B**) on the prosthetic side compared to the untuned condition but does not alter metrics on the sound side or change overall prosthesis or sound side leg work (**C**). The vertical and AP impulse symmetry indices (**D**) improved with tuning of prosthesis compared to the untuned condition. Individually colored lines represent individual subjects. *indicates significant differences (*p *< 0.05) between the untuned and tuned conditions.

Tuning the prosthesis increased the prosthetic side positive ankle peak power (*t *= −3.79 *p *= 0.009) and ankle work (*t* = −4.33, *p *= 0.005), but did not increase the overall prosthetic side leg work (*t *= −1.92, *p *= 0.103) as depicted in Figure 2. No significant differences were observed between the tuned and untuned conditions for the sound side for ankle peak power and work (*t *= 1.75, *p *= 0.131 and *t *= 1.45, *p *= 0.198, respectively).

### Impulse symmetry

We found significantly reduced symmetry indices in the vertical (*t *= 3.97, *p *= 0.007) and AP planes (*t *= 3.62, *p *= 0.011) in the tuned condition compared with the untuned condition as depicted in Figure 2, indicating an increased symmetry in the generation of force impulse on the ground.

### Limb-force alignment

**Figure 3:**
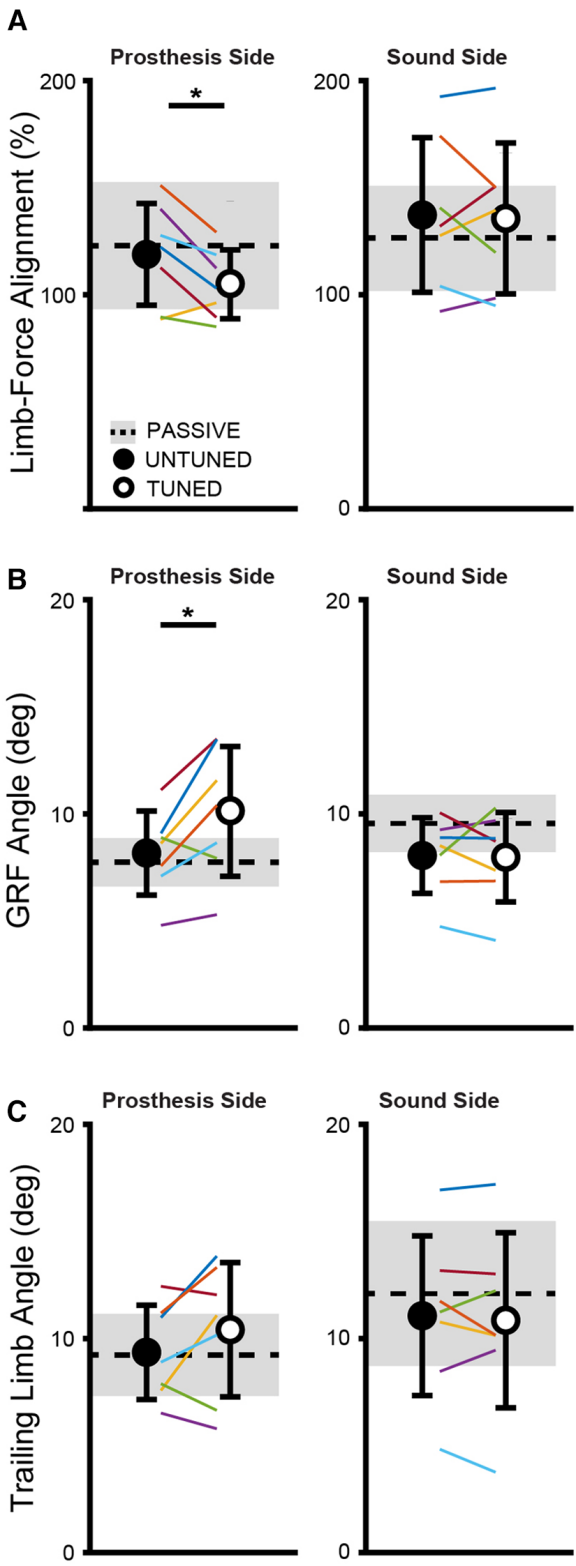
The tuned condition shows significantly enhanced alignment of the limb-force alignment (**A**) on the prosthetic side, which is the alignment between the angle of the sagittal plane ground reaction force (**B**) and the trailing limb angle (**C**). Changes in the limb force alignment are attributed to changes in the ground reaction force angle (**B**) and not the trailing limb angle (**C**). Individually colored lines represent individual subjects. *indicates significant differences (*p* < 0.05) between the untuned and tuned conditions.

An increased alignment was observed in the limb-force alignment with tuning compared with the untuned condition on the prosthesis side (*t *= 2.96, *p *= 0.025) as depicted in Figure 3. These changes are attributed to the changes in the ground reaction force angle measured on the prosthetic side that was directed significantly more anteriorly (*t *= −2.92 *p *= 0.027) in the tuned condition compared with the untuned condition. No significant differences were found in the prosthetic side trailing limb angle between the tuned and untuned condition (*t *= −1.49, *p *= 0.186). No changes were observed in the limb-force alignment on the sound side with tuning (*t *= 0.31, *p *= 0.768).

### GQI, POGS, and lateral sway

**Figure 4:**
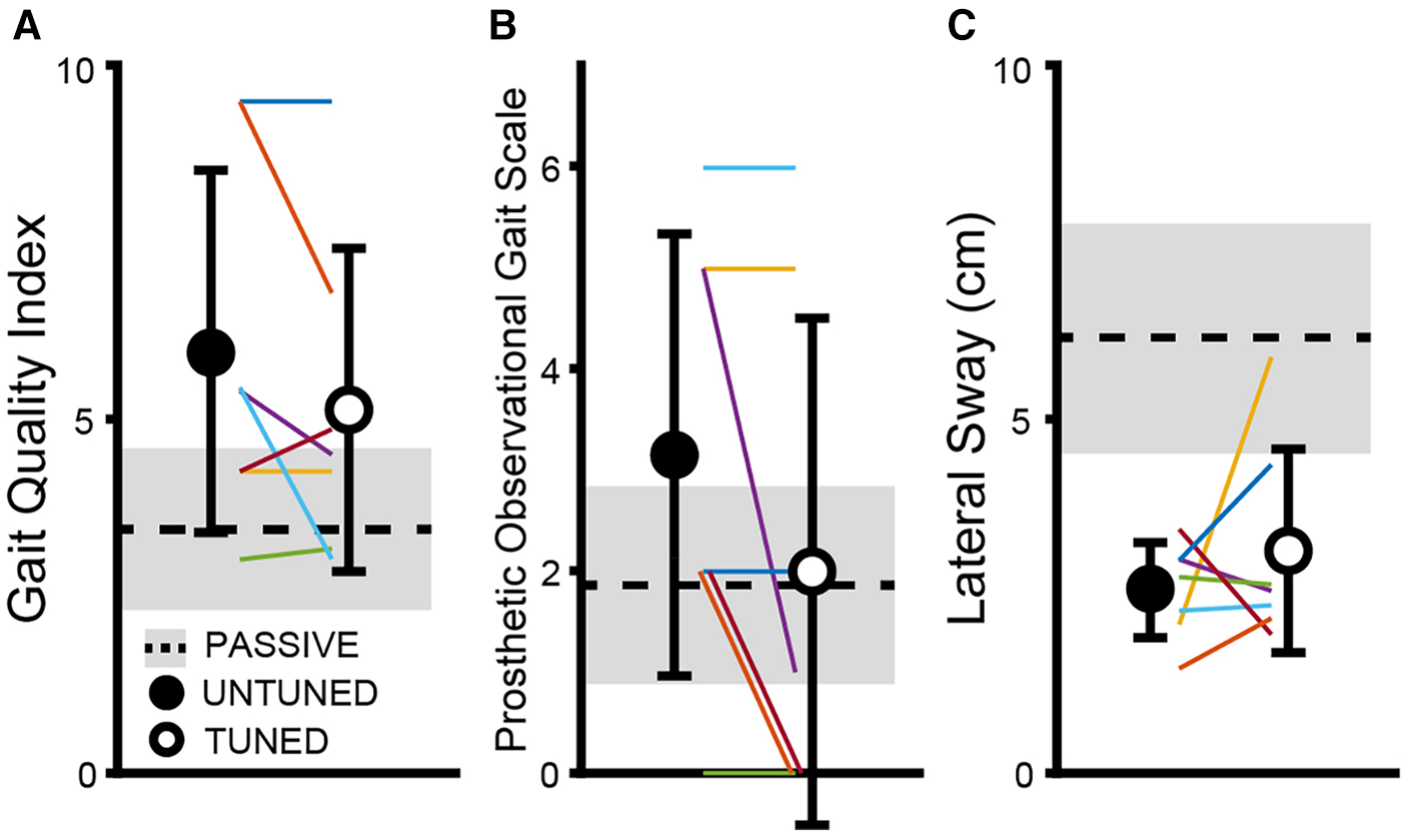
No significant differences are observed between the untuned and tuned conditions for the GQI (**A**), POGS (**B**,**C**), and lateral sway metrics. Individually colored lines represent the individual subjects.

As depicted in Figure 4, no significant differences were seen between the tuned and untuned conditions for GQI (*t *= 1.52, *p *= 0.18), POGS (*t *= 1.92, *p *= 0.103), and lateral sway (*t *= −0.01, *p *= 0.993) between the tuned and untuned conditions. Cadence, step and stride lengths, and step width are provided for the untuned and tuned conditions in Supplementary Table S2.

## Discussion

The tuning of a powered prosthetic foot is an iterative process that involves significant collaboration between the treating clinician and the patient. A clinician can visually assess a user's gait but also must be able to listen and translate a patient's perceptions into meaningful changes in the mechanical function and behavior of the prosthetic foot. The process is subjective, involves tradeoffs between the patient and clinician, and can necessitate a large amount of trial and error. This process may be exhausting and frustrating for a user and challenging for new and/or time-restricted clinicians. We investigated the response of different gait parameters as we tuned a research-grade powered prosthetic foot to see if any gait metrics could potentially be implemented in the future for a real-time, objective feedback during the tuning process to streamline the process for clinicians and patients alike.

Our data show an increase in positive ankle work and power, which indicates an improvement in the push-off capability following the tuning procedure, which was expected given that some of the parameters tuned by the research team are intended to affect the push-off power (e.g., max torque, min torque plantar). Notably, the peak prosthetic ankle power and work in the tuned condition become more similar in value to the sound side peak ankle power and work, indicative of more normative ankle kinetics after the tuning procedure. Improvements were also seen in the vertical and anterior–posterior impulse symmetry indices as well as the limb-force alignment metrics with the tuned condition compared with the untuned condition. Several studies have shown asymmetrical loading patterns between the sound and prosthetic limbs in individuals with amputation (37–39), and asymmetrical loading during walking may be linked to osteoarthritis and associated pain (40). Interestingly, both impulse symmetry and limb-force alignment relate to the timing of force generation during walking, which is information not traditionally accessible to a clinician during the prosthetic tuning process. The timing of force generation is critical when tuning powered prostheses; if the plantarflexion torque is delivered at the proper time, it will act to push the user forward, allowing the user to convert the external assistance into forward propulsion (41). In contrast, if delivered at the wrong time, the torque may instead push the user upward and/or lead to walking instability. Access to the force timing data through the use of future technology, such as wearable sensors, could allow for several benefits during the tuning process, including more normative biomechanics on the prosthetic side as well as increased symmetry between limbs. In addition, access to these data in a real-time clinical setting may speed up the tuning process by allowing for fewer tuning iterations.

Despite improvements seen in prosthetic side ankle work with the use of the tuned prosthetic foot, we found no improvements in overall prosthetic side leg work. This could be attributed to the loss of work within the prosthetic side knee and hip and the relatively short period of time in which participants wore the research-grade powered prosthetic foot, which was a limitation in this study. Prior work has shown the importance of the coordination of the human body and machine interface when a patient is interacting with a powered prosthetic foot (1), and our data suggest a reduction or tradeoff in prosthetic side knee power occurring during the tuned prosthetic foot condition (see Supplementary Figure S1). Further, the literature is highly varied in the reported duration of acclimation (42–44) required for a new prosthesis, and additional time may be necessary when transitioning from a passive system to a powered system. Our acclimation time to each parameter change in this study was relatively short (less than 1 min); however, because each change was performed iteratively, the participants had walked on the foot for approximately 30 min when the final-tuned condition was selected. The participants in this study, who were all experienced users of passive prosthetic feet, may have benefited from the additional acclimation time to adequately harness the push-off power provided by the powered prosthetic foot. Along with acclimation time, an additional limitation of this study is that the research team included a single prosthetist; a more clinically diverse research team (i.e., additional health professionals such as physical therapists and physiatrists) may have reached a different optimally tuned condition.

Our other metrics of gait quality (GQI, POGS, and lateral sway) are largely influenced by an individual's kinematics rather than kinetics and showed no significant differences between the untuned and tuned conditions of the powered prosthetic foot. The POGS is a clinical outcome measure that was initially developed in 2010 with the intent of providing clinicians with a more objective means for analyzing the changes in the overall gait. It includes visual assessment from the head (vaulting) and arms (arm swing) to all the joints of the lower extremities (36). However, there is no scoring aspect of the POGS that captures the differences between the powered and passive prosthetic feet—specifically, the ability to provide push-off assistance at terminal stance and varying range of motion (i.e., plantarflexion range of motion) and support provided during the mid- to late-stance transition. Although POGS evaluates the first ankle rocker of gait, it does not assess the second or third ankle rocker—key motions in powered prosthetic foot technology. In addition, some gait deviations assessed by POGS, such as vaulting and swing phase whip, may be less related to the tuning of a prosthetic foot and may instead be more reflective of learned habits or prosthesis alignment issues and thus not influenced by either good or bad tuning of a powered foot. Therefore, POGS may be inadequate to assess the nuanced changes in gait with the use of a powered prosthesis; updating the POGS outcome measure to include the assessment of the changes in gait that are common with the use of powered prosthetic technology may allow for greater discrimination during the use of varying powered technology. Alternatively, combining a secondary metric with the POGS that is sensitive to push-off kinetics may augment the POGS when assessing gait with the use of powered technology (45). The overall higher mobility level of the participants in this study may have influenced the lack of changes observed in lateral sway, POGS, and GQI. All participants were highly active, K3–K4 level walkers; this level of ability may allow them to adapt more readily to forced changes in their walking, particularly as it relates to kinematics. The task prescribed to the subjects was relatively simple—the subjects walked at a fixed speed constrained by the dimensions of the treadmill—so the difficulty associated with this task may not have been enough to impact the lateral sway (46). Further, all participants were able to maintain this walking speed in a safe manner throughout the duration of the trial even though the trajectory from the untuned condition to the final-tuned condition did not occur in a linear manner (i.e., some parameter changes made the performance worse). Notably, even when participants encountered parameter changes that were uncomfortable, all participants were able to continue walking.

Overall, our data show changes in the peak power, impulse symmetry, and LFA following the tuning procedure, forcing us to reject our hypothesis that more comprehensive metrics, such as GQI and POGS, would be able to detect changes following the tuning of a powered prosthetic foot. It is instead the metrics that focus on a single limb or the ankle joint alone that best reveal changes between the untuned and tuned conditions.

Our study is limited by several factors, including a relatively small sample size; a larger participant pool may have potentially shown some additional metrics to be of significant use for reflecting differences between the two conditions. Given that our selected foot is a tethered design, the tuning procedure was limited to the treadmill. Therefore, the results herein may not translate to tuning procedures conducted clinically, which typically occur in overground settings (47, 48). Further, we did not measure self-selected walking speed in the clinically prescribed prosthesis and required all participants to walk at a speed of 1.0 m/s for all trials. This set speed may have impacted our results and prevented the participants from naturally increasing their self-selected walking speed with the added push-off power from the device. While we had hoped that our selected clinical measure, the POGS, would reveal differences between the untuned and tuned conditions, it was not sensitive enough to show differences between the two conditions. An additional clinical outcome such as the 2-min walk test or timed up and go test conducted at the baseline and final-tuned condition may have shown clinically meaningful improvements; however, the tethered nature of the foot prevented our ability to conduct such tests. Because of this, we are forced to rely on the biomechanical metrics (positive ankle work and power), but these metrics may not necessarily reflect true improved clinical outcomes. We therefore relied on user feedback and clinical judgment throughout the procedure, which mimics common clinical practices; however, common clinical practice standards would greatly benefit from the administration of clinically meaningful outcomes before and after tuning these types of devices. Importantly, the results shown here are focused on biomechanical optimization of tuning; further research is needed to understand if these improvements translate to improvements in other performance-based and patient-reported outcomes. The results shown here, if operationalized in the form of wearable sensors that provide and/or fuse kinematic and kinetic data in real-time, could underscore the value of the expertise required for tuning a prosthesis and may facilitate changes to current reimbursement practices. In addition, we selected a single walking speed of 1.0 m/s, which falls within previously reported values for transtibial prosthesis users during level walking (24) to ensure that our participants would be able to maintain the speed throughout the duration of the study. However, testing of additional higher speeds may have revealed more positive outcomes in some of our other selected metrics, especially given that some evidence suggests that walking speeds can increase with the use of powered feet (3). Finally, familiarity with the research-grade prosthetic foot over the length of the trial may have influenced the outcomes, and a repetition of the initial baseline untuned trial after the final-tuned trial would have allowed for comfort and familiarity to be removed as a variable associated with the final-tuned trial.

Interestingly, the device was tuned in this study through standard clinical methods, which only include observational gait analysis and participant verbal feedback. Despite the lack of real-time biomechanics data, significant improvements were noted in several metrics related to force timing. However, as previously noted, these improvements took approximately 24 iterations of parameter tuning to achieve. Our results suggest that this force timing information may be impactful in aiding clinicians in helping their patients achieve a more biomechanically normative and symmetrical gait. In addition, a real-time provision of this data in the future through the use of wearable sensors may augment the ability of a clinician to tune a prosthesis for an individual patient with greater ease and speed than relying on current methods alone.

## Conclusion

Our expectation is that this work may extend beyond applications of powered feet in users with transtibial amputation and may also be useful during the prosthetic fitting process for users with transfemoral amputation as well as more commonly prescribed passive devices. The prescription and selection of prosthetic components, as well as the alignment process, are critical aspects for long-term user success and comfort. Indeed, it is known that the alignment of prosthetic components can have an influence on a patient's metabolic cost (11), their overall comfort within the prosthetic socket (49), and, more importantly, their gait and posture (17, 18). Relevant data provided to the prosthetist can enhance and improve the current clinical process associated with the fitting and delivery of prostheses, as well as their long-term outcomes. The metrics detailed herein are not exclusively designed for usage with powered devices and could be used to enhance the prosthetic tuning process and the overall clinical outcomes for patients.

## Data Availability

The raw data supporting the conclusions of this article will be made available by the authors, without undue reservation.

## Supplementary material

Supplementary Figure S1 UDAF, knee, hip, and leg power traces are shown for a representative subject for the prosthetic and sound sides.
