# Supplementary figures and images for: Gait quality in prosthesis users is reflected by force-based metrics when learning to walk on a new research-grade powered prosthesis
Source: Front Rehabil Sci. 2024 Feb 2;5:1339856. doi: 10.3389/fresc.2024.1339856 (PMC10869520; doi:10.3389/fresc.2024.1339856)

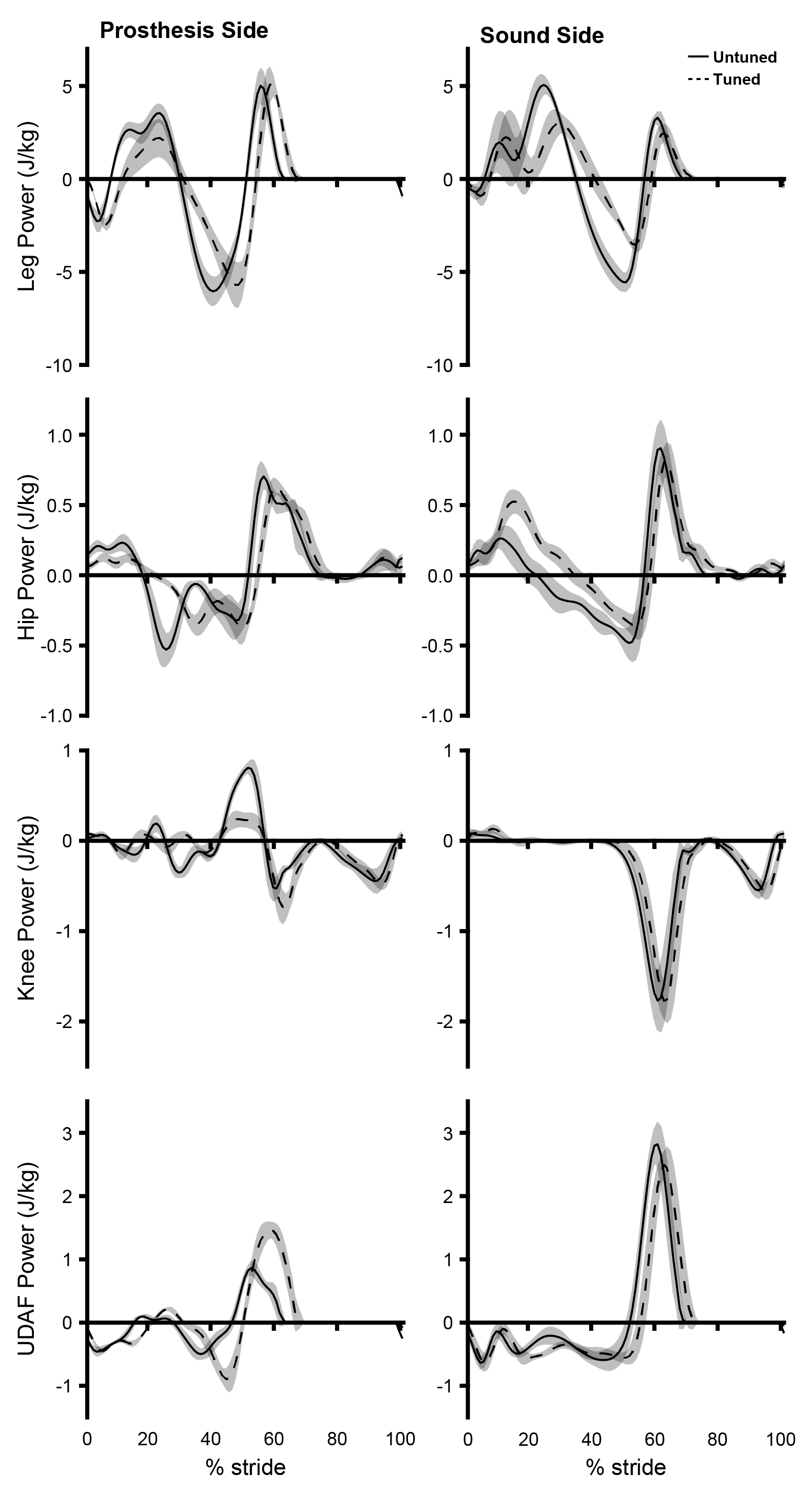

Supplement: Supplementary Figure S1 — UDAF, knee, hip, and leg power traces are shown for a representative subject for the prosthetic and sound sides. [file Image1.jpg]
